# Clinical Correlation of Retinal Fluid Fluctuation Represented by Fluctuation Index in Wet Age-Related Macular Degeneration: TOWER Study Report 2

**DOI:** 10.1167/tvst.12.10.2

**Published:** 2023-10-03

**Authors:** Yodpong Chantarasorn, Paisan Ruamviboonsuk, Somanus Thoongsuwan, Sritatath Vongkulsiri, Pavinee Kungwanpongpun, Prut Hanutsaha

**Affiliations:** 1 Department of Ophthalmology, Vajira Hospital, Navamindradhiraj University, Bangkok, Thailand; 2 Department of Ophthalmology, Rajavithi Hospital, Rungsit University, Bangkok, Thailand; 3 Department of Ophthalmology, Siriraj Hospital, Mahidol University, Bangkok, Thailand; 4 Department of Ophthalmology, Phramongkutklao Hospital, Phramongkutklao College of Medicine, Bangkok, Thailand; 5 Medical Affairs, Novartis (Thailand) Ltd., Bangkok, Thailand; 6 Department of Ophthalmology, Ramathibodi Hospital, Mahidol University, Bangkok, Thailand

**Keywords:** retinal fluid fluctuation, macular neovascularization, fluctuation index, anti-VEGF, Thailand

## Abstract

**Purpose:**

To explore outcomes and biomarkers associated with retinal fluid instability represented by a new parameter in neovascular age-related macular degeneration (nAMD).

**Methods:**

Patients with treatment-naïve nAMD receiving anti-vascular endothelial growth factor (VEGF) injections for a duration of 1 to 3 years were consecutively reviewed. Fluctuation Index (FI) of each eye, calculated by averaging the sum of differences in 1-mm central subfield thickness between each follow-up from months 3 to 24, was arranged into ascending order from the lowest to the highest and split equally into low, moderate, and high fluctuation groups. Outcomes were analyzed at 24 months.

**Results:**

Of 558 eyes, FI values showed a negative correlation with a degree–response gradient with 24-month visual improvement. After controlling for baseline best-corrected visual acuity and potential confounders, eyes with low fluctuation gained more Early Treatment Diabetic Retinopathy Study letters than those in the moderate and high fluctuation group (Δ, 10.1 and 14.0 letters, respectively). Significant best-corrected visual acuity improvement from baseline to month 24 (11.8 letters) was observed exclusively in the low fluctuation group despite the indifference in the number of injections and types of anti-VEGF drug used among groups. Patients presenting with central subfield thickness of ≥405 µm or intraretinal fluid coinciding with subretinal fluid showed a significant association with foveal thickness instability during the maintenance phase.

**Conclusions:**

Apart from the central subfield thickness values, unstable macular thickening represented by the FI was associated with some baseline features and may contribute to substandard visual outcomes.

**Translational Relevance:**

FI may be a valuable tool for assessing therapeutic adequacy in the treatment of nAMD.

## Introduction

In clinical practice, undertreatment with intravitreal anti-vascular endothelial growth factor (VEGF) injections appear to be a key factor for continuing visual acuity loss in the treatment of neovascular age-related macular degeneration (nAMD).^1^^,^^2^ These observations have been supported by results from the Treatment Outcome of Wet Age-Related Macular Degeneration Management in Thailand (TOWER) study,^3^ describing real-world outcomes of nAMD treatment in Thai patients, where polypoidal choroidal vasculopathy (PCV) was a predominant feature (58%), and bevacizumab (Roche, Basel, Switzerland) was selected as the first-line medication based on the national health policy. The study found that the number of injections declined substantially after 24 months. This issue led to a loss of visual benefit at 36 months.^3^ Using parts of a similar dataset, this extension study primarily analyzed association of retinal fluid variability with treatment outcomes, and risk factors for its development.

Inadequate suppression of retinal fluid, particularly intraretinal fluid (IRF), has resulted in poor visual outcomes despite continuous anti-VEGF treatment.^4^ However, poor visual outcomes may stem not only from the persistency of retinal fluid, but also from unstable macular thickness over the treatment course.^5^ In particular, anti-VEGF medications achieve high levels of vitreous concentration during the early phase, and gradually decrease over the interval between injections. The rate of drug clearance depends not only on each patient's vitreoretinal morphology or medication tachyphylaxis, but on nuances in drug ,properties including half-life and binding affinity.^6^^,^^7^ Combined with variations in disease activity, these factors result in fluctuations in foveal thickness, especially in the real-world setting where the treatment intervals may not be as consistent as those in most clinical trials, which frequently have a minimum duration of 24 months.^3^^,^^8^^,^^9^ Hence, retinal fluid fluctuation from month 3 to month 24 can be viewed as an indirect, yet simple tool to assess macular neovascularization (MNV) activity and individualized responses after a course of anti-VEGF treatment.^5^^,^^10^^–^^12^

A parameter measuring fluctuations in foveal thickening is not obtained in a straightforward manner from imaging devices, but rather requires a calculation using retinal thickness values pooled from several treatment visits. Most clinical trials defined a magnitude of macular thickness variation by the standard deviation (SD) of central foveal thickness after three loading doses.^5^^,^^13^ From a clinical practice standpoint, we constructed a simulated model to demonstrate an example of variable patterns of changes in macular thickness and their corresponding SD of retinal thickness. Interestingly, the chart showed that a patient with a clear zigzag pattern of retinal thickness has the lowest SD value (Supplementary Fig. S1). We, therefore, proposed a new parameter called the Fluctuation Index (FI) to represent fluctuating degrees of retinal fluid. This parameter was modified from the Fluctuation Score reported in a post hoc analysis of the Study of Ranibizumab Administered Monthly or on an As-needed Basis in Patients With Subfoveal Neovascular Age-related Macular Degeneration (HABOR) study.^14^ We also investigated to determine whether FI would be a superior method than traditional central subfield thickness (CST) SD to illustrate a relationship between visual results and foveal thickness fluctuation.

Apart from the inconsistency of treatment intervals, little is known about factors associated with macular fluid volatility, specifically factors that can be used in daily practice. Therefore, this study aimed to determine predictive biomarkers associated with macular thickness fluctuation during nAMD treatment and to explore the impact of retinal fluid fluctuation, represented by the FI, on visual outcomes.

## Methods

This retrospective cohort analysis was conducted at five university hospitals in Thailand from January 2016 to December 2018. The study was approved by the Central Research Ethics Committee (COA No. 095/2020) and performed in accordance with ethical principles based on the Declaration of Helsinki.

The TOWER Study consecutively enrolled treatment-naive patients with nAMD who underwent anti-VEGF injections for a period between 12 months and 3 years. The study included patients aged >40 years who received treatment starting from January 2016 to December 2018, allowing for one missing visit with a maximal follow-up interval limited to 6 months. This extension study only included those who had completed 24 months of follow-up. One eye per patient was included to avoid any confounding effects resulting from codependent variables. Only the worse-seeing eye was enrolled if the patient had bilateral nAMD. All patients must have had at least one fundus fluoresceine/indocyanine green angiography or optical coherence tomography (OCT) angiography, and complete OCT scans using Spectralis OCT (Heidelberg Technology, Heidelberg, Germany) at every visit. Other preexisting retinopathies, such as macular atrophy, subretinal fibrosis, myopic MNV, chorioretinitis, diabetic retinopathy, and retinal vascular occlusions, were excluded from the study. We also excluded type III MNV owing to small case numbers and the more aggressive nature of leakage in the neurosensory retina. Patients undergoing intraocular surgery, macular laser, or photodynamic therapy during the study period were not enrolled. Retinal fluid was categorized into three subtypes: IRF (fluid located inside the neurosensory retina), subretinal fluid (SRF or fluid residing in the subretinal space), and pigment epithelial detachment (PED), which was coded when serous components were present underneath the elevated retinal pigment epithelium. Drusenoid PED was excluded for the analysis. Other standard ocular parameters were reviewed at every visit after the initiation of anti-VEGF treatment.

Treatment decisions were primarily determined by the physicians’ (five authors) assessment. In general, any amount of SRF or IRF would be promptly treated based on a treat-and-extend protocol initiated with three monthly bevacizumab injections,^15^ while either central foveal thickness or the occurrence of PED had a much lesser role on the treatment decision compared with the OCT morphology. In particular, shallow PED unaccompanied by SRF or IRF was likely tolerated if it remained unchanged over the treatment course. All patients were required to initiate treatment with bevacizumab for a minimum of three monthly injections before contemplating a switch to branded medications. We designed a questionnaire to assess the practice patterns of all authors with respect to drug regimen switching. For eyes without polypoidal lesions, the physicians frequently switched from bevacizumab to branded anti-VEGF drugs when there was an increase or persistency in SRF or IRF after 3 to 6 consecutive bevacizumab injections, regardless of visual acuity levels. However, in patients with PCV, persistence of polypoidal lesions seen on indocyanine green angiography or steep-angled PED coinciding with double-layered signs on OCT scans was additionally taken into the switching criteria despite the absence of IRF or SRF.

We extracted data from each of all eligible cases to calculate the FI: a sum of differences (decreased or increased) in 1-mm CST between each follow-up visit during the maintenance phase from the completion of initial anti-VEGF loading (month 3) to month 24, divided by the total number of visit intervals (the total number of visits = 1). The index values were organized into ascending order from the lowest to the highest and, thereafter, split equally into three groups: low, moderate, and high CST fluctuation groups. CST values during the loading phase were omitted from the calculation because a sharp decrease in the retinal fluid amount could lead to large variability in the CST that may outweigh the index value of each patient's CST during their postloading phase.^13^^,^^14^ We propose that FI should offer suitability for our real-world study primarily owing to inconsistent follow-up intervals.

To validate the potential application of the new parameter in this study, the correlations between the two fluid fluctuation metrics—CST SD and FI versus best-corrected visual acuity (BCVA) improvement—was analyzed at the 24-month visits. Concurrently, anatomic, and visual results at months 24 in each group, were computed using generalized estimating equations (GEEs).

Patients with a thick CST at the initial setpoint will likely have a sharp decrease in retinal fluid amount resulting in a greater CST decrease after receiving bevacizumab initiation, compared with those whose baseline CST is close to their physiological foveal thickness. Thus, the CST values need to be controlled when assessing predictors of CST fluctuation.^10^^,^^12^ In this study, a receiver operating characteristics (ROC) curve was performed to calculate the optimal cut-off point of baseline CST with the best sensitivity and specificity for detecting unstable CST after initial loading of anti-VEGF injections. To determine its odds ratio, this value was thereafter applied as one of the potential factors associated with high CST fluctuation in the first multivariate logistic regression model.

A second logistic regression model, adjusted for age and baseline CST, was performed to identify retinal fluid subtypes predicting high CST variation. Because each study eye could possess mixed subtypes of retinal fluid, the presence of SRF combined with IRF, and PED combined with SRF or IRF at the baseline visit were additionally considered as clinically important factors in the model.

Notably, the use of bevacizumab was considered one of the factors included in the first logistic regression model that aimed to determine risk factors for retinal fluid fluctuation. In particular, the first model analyzed the use of bevacizumab in all patients, including those receiving a switched regimen with a minimum of three loading injections of bevacizumab. To explore the exclusive effects of each anti-VEGF agent as the secondary outcomes, we separately performed additional analyses of retinal fluid fluctuation metrics and clinical outcomes, categorized by each anti-VEGF drug, exclusively in patients receiving the same anti-VEGF agent after the loading phase.

### Statistical Analysis

Skewed data were logarithmically transformed before analysis. Regarding the BCVA analysis, a global comparison of the three mean differences in letter gain was tested using one-way analysis of variance considering *P* values of <0.05 as statistically significant. If significant differences were detected, a post hoc pairwise comparison was performed using GEE. After validation of the implemented model, GEE controlling for age, baseline retinal thickness, baseline BCVA, a total number of aflibercept injections, and a total number of injections was performed to detect differences in mean 24-month BCVA changes in each pairwise comparison. The comparison was not performed at months 3 and 12 because the FI was derived from patients later in time.

A multiple logistic regression model controlling for age, baseline BCVA, use of bevacizumab, and a diagnosis of PCV, was performed to identify potential factors associated with a highly fluctuating macular thickness over the treatment course. Other statistical tests used are described in the footnotes of each table. All missing time points were not handled by statistical tools. A two-sided *P* value of <0.05 was considered statistically significant. Stata version 15.0 (StataCorp, College Station, TX) was used for all computations. All diagrams and charts were constructed by SAS version 9.4 (SAS Institute, Cary, NY).

## Results

Of the 558 eligible eyes, the annual visit numbers per one case were 9.1 ± 0.2 and 7.4 ± 0.4 in the first and second years of treatment, respectively. The SD of the CST during the first and the second years of treatment seemed to be underestimated when compared with the mean values derived from the FI method, especially in the high fluctuation group (102 vs. 120 and 104 vs. 117 at 12 and 24 months, respectively), This finding seemed to align with the schematic illustration depicted in Supplementary Figure S1 (case 3). Both CST SD and FI values seemed to be consistent from month 12 to month 24 in the moderate fluctuation group (Table 1).

**Table 1.** Demographic and Clinical Characteristics of Patients With Neovascular AMD Categorized by the SD of 1-mm CST From Month 3 to Month 24

					Pairwise Comparisons		
					Low vs Moderate Fluctuation	Low vs High Fluctuation	Moderate vs High Fluctuation
	Degree of Fluid Fluctuation						
Results	Low (186 Eyes)	Moderate (186 Eyes)	High (186 Eyes)	Global *P* Value	Mean Differences (95% CI), *P* Value		
Age (years)	68.1 ± 10.1	68.0 ± 10.6	69.7 ± 10.0	0.26^a^			
Male, n (%)	94 (50.5%)	111 (59.7%)	103 (55.4%)	0.38^b^			
CST (µm)^c^					–56.5 (–108 to –4.2) 0.03	–192 (–242 to –141) **<0.0001**	–135 (–184.7 to –86.0) **<0.0001**
Baseline	324 ± 114	380 ± 155	515 ± 213	**<0.0001** **^d^**			
Month 3	253 ± 67	296 ± 113	383 ± 165	**NA**			
Month 12	251 ± 65	280 ± 93	382 ± 194	**NA**			
Month 24	248 ± 54	274 ± 72	407 ± 178	**<0.0001** **^d^**	–25.4 (–77.3 to 26.5) 0.71	–160 (–213 to –105) **<0.0001**	–134 (–186 to –81.5) **<0.0001**
FI (months 3–12)	14.6 ± 7.2	42.9 ± 9.8	120.5 ± 57.6	**<0.0001** ^a^			
SD of CST (months 3–12)	13.9 ± 10.9	40.0 ± 22.6	102.0 ± 70.8	**<0.0001** ^a^			
FI (Months 3–24)	15.5 ± 7.6	42.8 ± 9.1	117.8 ± 60.3	**<0.0001** ^a^			
SD of CST (months 3–24)	11.8 ± 6.3	40.6 ± 10.6	104.7 ± 59.3	**<0.0001** ^a^			
Baseline logMAR BCVA^3^	0.73 ± 0.52	0.78 ± 0.61	1.03 ± 0.55	**<0.0001** ^a^	–0.05 (–0.19 to 0.08) 1.0^c^	–0.30 (–0.44 to –0.16) **<0.0001****^c^**	–0.24 (–0.38 to –0.10) **<0.0001****^c^**
ETDRS letters gain Year 1^e^ (95% CI compared with baseline), *P* value	9.9 (7.2 to 12.6), **<0.0001**	5.24 (2.1 to 8.3), **0.0008**	1.7(–1.9 to 5.4), 0.35	NA			
Year 2^e^ (95% CI compared with baseline), *P* value	11.8 (6.6–17.1), **<0.0001**	4.3 (–0.8 to 9.4), 0.10	3.2 (–3.6 to 10.2), 0.35	0.40^d^	10.1 (1.3 to 21.4) **0.05**	14.0 (2.9 to 25.0) **0.01**	3.9 (–7.3 to 15.2) 0.40
Log MAR BCVA at year 2	0.46 ± 0.36	0.66 ± 0.50	0.98 ± 0.61	**<0.0001** **^d^**	–0.20 (–0.40 to –0.01) 0.05	–0.51 (–0.72 to –0.30) **<0.0001**	–0.32 (–0.52 to –0.11) **0.0008**
PCV, n (%)	104 (55.91%)	106 (56.99%)	115 (61.83%)	0.87			
Eyes receiving bevacizumab (%)	139 (74.73%)	147 (79.03%)	153 (82.26%)	0.31			
No. of injections (months 3–24)	8.0 ± 5.2	8.5 ± 4.7	7.6 ± 4.6	0.11^d^			

ETDRS, Early Treatment Diabetic Retinopathy Study.

a *P* value based on the Kruskal-Wallis test.

b Two-sided *P* value based on the χ^2^ test.

c *P* value for each pairwise comparison based on the Bonferroni post hoc test.

d A global *P* value based on one-way analysis of variance.

e The ETDRS letter gain controlled for age, baseline retinal thickness, baseline BCVA, a total number of aflibercept injections, and a total number of anti-VEGF injections was calculated based on GEEs.

For each timepoint comparison, multivariate linear regression adjusted for the same covariates was performed.

In this cohort, ≤40% of patients were switched to branded drugs consisting of aflibercept (35.2%) and ranibizumab (4.9%) after having been treated with bevacizumab for a mean duration of 9.0 ± 8.5 months. Regarding the overall numbers of anti-VEGF injections, bevacizumab, aflibercept (Bayer, Leverkusen, Germany) and ranibizumab (Novartis, Basel, Switzerland) accounted for 78.5%, 17.1%, and 4.3%, respectively.^3^

No significant differences were detected in the injection number of each anti-VEGF drug used per patient or the total numbers of anti-VEGF injections observed among the three groups at the second year of treatment (Table 2). Of the patients who had a diagnosis of PCV or MNV in conjunction with pachychoroid diseases, 21 eyes (3.7% of the study patients) were under the age of 50. In addition, no differences in terms of proportions of eyes diagnosed with PCV and those receiving bevacizumab were observed among the three study groups (Table 1).

**Table 2.** Twenty-Four–Month Injection Numbers for Each Anti-VEGF Categorized by SD of CST From Month 3 to 24

Total No. of Injections Over 2 Years (Mean ± SD)				
Characteristics	Low Fluctuation (*n* = 186)	Moderate Fluctuation (*n* = 186)	High Fluctuation (*n* = 186)	Global *P* Value^*^
Bevacizumab	4.9 ± 3.0	5.7 ± 3.3	5.4 ± 3.5	0.07
Ranibizumab	2.7 ± 2.0	2.5 ± 3.0	2.7 ± 3.0	0.25
Aflibercept	4.6 ± 3.0	5.8 ± 3.9	5.4 ± 3.8	0.15

* *P* value based on the Kruskal-Wallis test.

Based on the correlation analysis, although not statistically significant, there seemed to be a gradual downward trend in visual improvement as the FI increased. At month 24, the associations between the two variables were low to moderate, with Pearson's correlations (rho) of −0.16 (*P* = 0.49). Further analysis of patients’ subgroups revealed increasing magnitudes of the correlations from the low to the high fluctuation group (rho, −0.048 to −0.28, respectively). In contrast, the CST SD values demonstrated a weak but positive correlation (rho, 0.07; *P* = 0.45) with letter improvement at 24 months; analogously, the spread of such rho values increased from the low to the high fluctuation group (rho, −0.06 to 0.34, respectively), but in the opposite direction (Table 3).

**Table 3.** Pearson's Correlation Coefficient Between Different Fluid Fluctuation Metrics and ETDRS Letter Gain in All Study Patients Who Received the Same Anti-VEGF Agents During the 24-month Treatment Period

Results	All Patients	Low Fluctuation	Moderate Fluctuation	High Fluctuation
CST SD^a^				
Month 24 (n)^b^	115 eyes	39 eyes	39 eyes	37 eyes
Correlation coefficient	0.07	–0.06	0.12	0.34
(95% CI)	(–0.15 to 0.21)	(–0.35 to 0.25)	(–0.21 to 0.43)	(0.021 to 0.60)
2-Tailed significance	0.45	0.71	0.46	0.03
FI^c^				
Month 24 (n)^b^	115 eyes	39 eyes	39 eyes	37 eyes
Correlation coefficient	–0.165	–0.048	–0.094	–0.28
(95% CI)	(–0.24 to 0.12)	(–0.36 to 0.27)	(–0.40 to 0.23)	(–0.30 to 0.035)
2-tailed significance	0.49	0.77	0.57	0.37

ETDRS, Early Treatment Diabetic Retinopathy Study.

a Patients were categorized equally into three groups, based on CST SD, that were organized from the lowest to the highest values.

b The number of analyzed patients was markedly reduced because data were solely extracted from those who did not receive a switched anti-VEGF therapy regimen.

c Patients were categorized equally into three groups, based on FI, that were organized from the lowest to the highest values.

At the baseline visit, the mean CST in the high fluctuation group (515 µm) was significantly greater than that in the moderate (380 µm) and low fluctuation groups (324 µm). However, all groups exhibited a trend towards CST reduction throughout the treatment course. At 24 months, no differences in CST were detected between the low and moderate fluctuation groups (*P* = 0.71) (Table 1, Supplementary Fig. S2).

**Figure 1.**
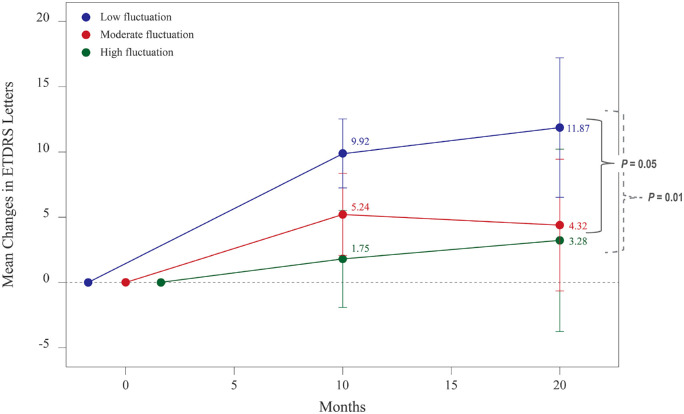
Plots of BCVA improvement based on GEEs. After controlling for age, baseline 1-mm CST, baseline visual acuity, and a total number of injections, the differences in the BCVA improvement between the low and high fluctuation groups had increased from 9.7 letters at 12 months to 14.0 letters at 24 months follow-up. The pairwise comparison with *P* values of ≤0.05 were depicted at 24-month follow-up.

Regarding visual outcomes, the calculation based on the GEE controlling for the covariates showed that patients in the low fluctuation group gained more Early Treatment Diabetic Retinopathy Study letters significantly than those in the high fluctuation groups (mean differences, 14.0 letters; 95% confidence interval [CI], 3–25 letters; *P* = 0.01) at 24 months (Fig. 1). When the comparisons were made within the same patient group, significant BCVA improvement from baseline to month 24 was observed exclusively in the low fluctuation group (11.8 letters; 95% CI, 6.7–17.1 letters; *P* < 0.0001). At study exit, eyes with a high CST fluctuation had significantly worse BCVA (mean Snellen equivalent, 20/200^−1^) than eyes with low (20/63^+2^) or moderate (20/100^+2^) CST fluctuation (Table 1). Using the similar model, when replacing FI with the values calculated from month 3 to month 12, mean 24-month BCVA improvement was 8.0 (95% CI, 2.6–13.3), 6.6 (95% CI, 1.2–11.9), and 4.2 (95% CI, −2.7 to 11.2) Early Treatment Diabetic Retinopathy Study letters in the low, moderate, and high fluctuation groups, respectively (*P* = 0.021). A subsequent model using C-statistic calculated from ROC analysis demonstrated that an FI of <27.8 would obtain the favorable sensitivity (68%) and specificity (60%) for detection of mean visual results observed in the low fluctuation group (0.48 [20/63^+1^] ± 0.34).

**Figure 2.**
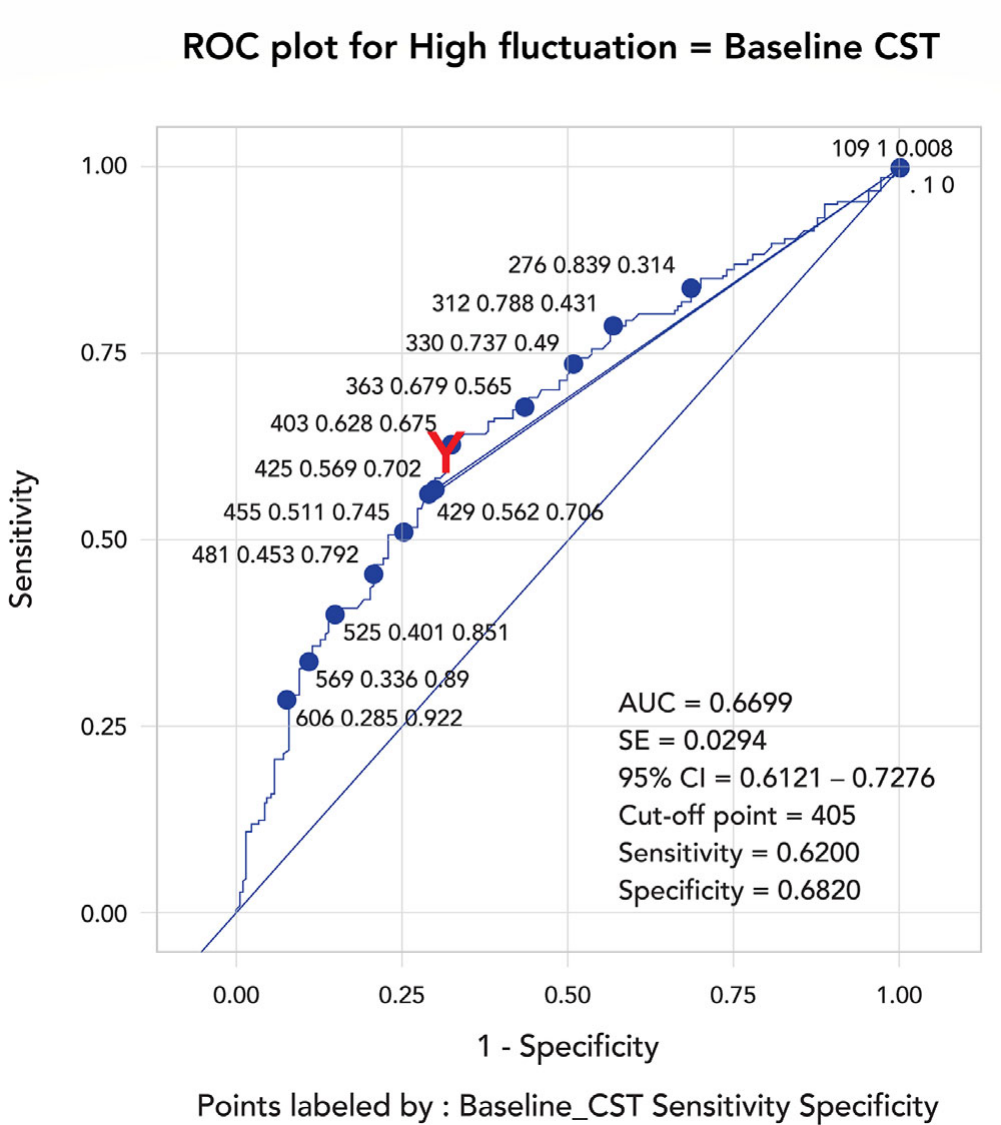
ROC curves show that baseline 1-mm CST above 405 µm have good predictability of highly fluctuating retinal fluid during month 3 to 24 after initiation of anti-VEGF treatment. The area under the ROC curve, sensitivity, and specificity of detection were 67%, 62%, and 68%, respectively.

Among baseline characteristics, an initial multiple logistic regression analysis showed that baseline CST analyzed as a continuous variable was the only factor significantly associated with highly fluctuating retinal thickness throughout 24 months. The ROC curve subsequently demonstrated that a baseline CST of ≥405 µm yielded the most favorable sensitivity and specificity of 62% and 68%, respectively (area under the ROC curve, 0.67) (Fig. 2). This value persistently showed high CST instability with an adjusted odds ratio of 3.27 (95% CI, 2.08–5.13) in the same multivariate analysis when the continuous variable of baseline CST was substituted by this cut-off point. No correlations were observed between highly fluctuating CST and the following variables: baseline BCVA, the presence of a polypoidal lesion, and bevacizumab use (Supplementary Table S1).

**Figure 3.**
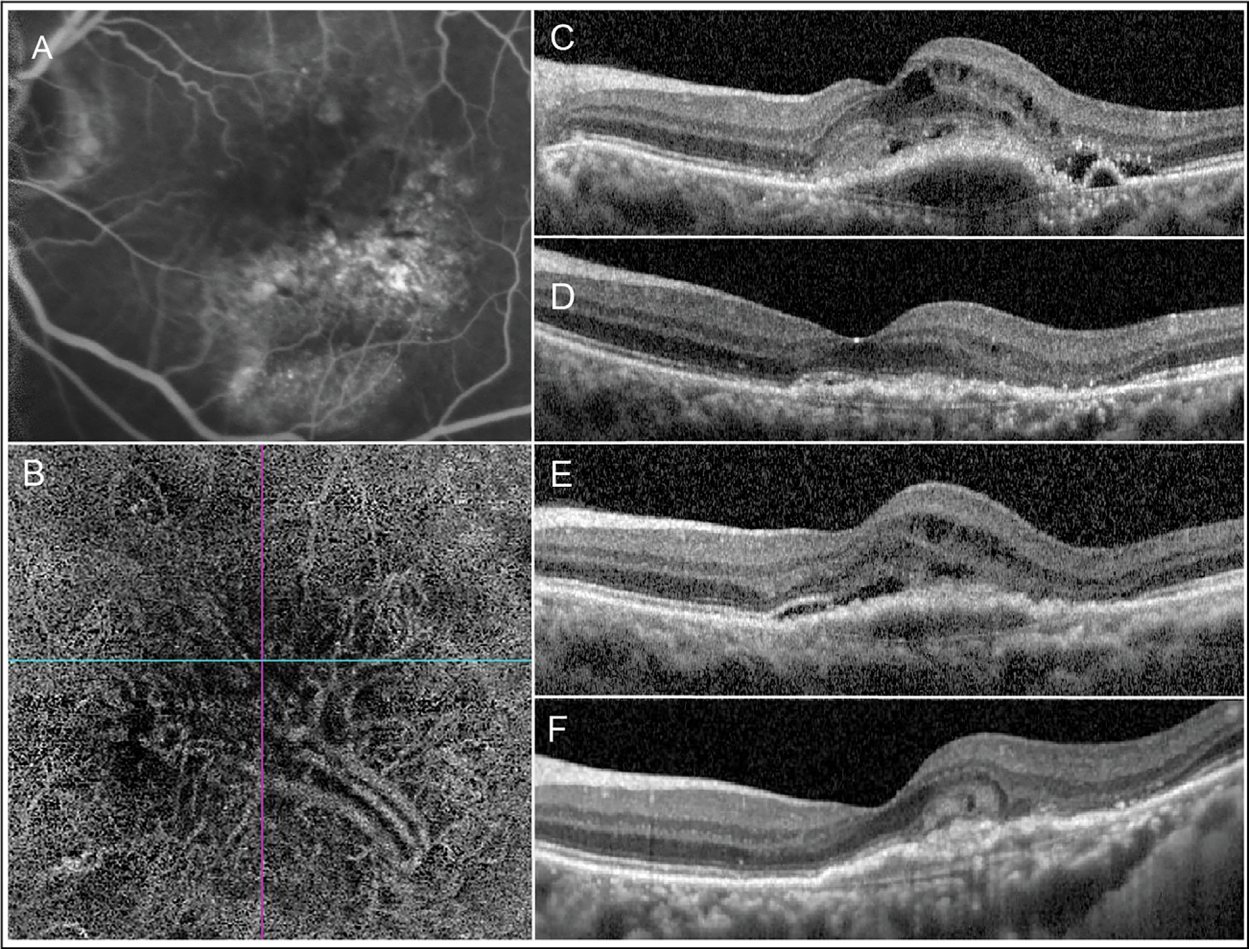
Multimodality imaging of a woman in her late 60s with type I subretinal neovascular membrane who received 23 injections of bevacizumab and 4 injections of aflibercept using treat-and-extend regimen over 38 months. (A) Fundus fluorescein angiogram showed occult leakage in the late venous phase without polypoidal lesions seen on indocyanine angiography. (B) OCT angiography disclosed a large area of neovascularization at the choriocapillaris level. (C) The presenting OCT scan showed IRF combined with subretinal fluid overlying the neovascular membrane, with 1-mm CST of 381 µm. (D, E) Retinal fluid had recurred almost every time the monthly injection interval was extended to 6 weeks. The CST values had been periodically fluctuated over 24 visits (month 3–24) with CST ranged from 246 to 423 µm and the SD and FI of 37.2 and 48.2 (a total fluctuation score, 1,157 µm), respectively. (F) At 3 years, there was an absence of retinal fluid with development of outer retinal tubulation and fibrotic scaring (CST, 314 µm); BCVA had changed from 20/100-2 at baseline to 20/50+1 and 20/80-1 at 1 and 3 years, respectively.

The second multiple logistic regression model, analyzing retinal fluid subtypes, showed that the presence of IRF with and without SRF at the baseline visit was significantly associated with fluctuating macular thickness over the treatment course (adjusted odds ratio, 2.10 and 1.96; *P* adjusted for baseline CST and age = 0.003 and 0.004, respectively) (Table 4, Fig. 3).

**Table 4.** Multiple Logistic Regression Identifying Retinal Fluid Subtypes Associated With Large CST Fluctuation from Months 3 to 24 in Eyes With Neovascular AMD

			OR			
Types of Retinal Fluid At the Baseline Visit	High Fluctuation (186 Eyes)	Low to Moderate Fluctuation (372 Eyes)	Crude OR (95% CI)	*P* Value	Adjusted OR^*^ (95% CI)	*P* Value^a^
SRF	125 (67.20%)	175 (47.0%)	1.75 (1.22–2.51)	0.002	1.40 (0.79–2.48)	0.24
IRF	77 (41.40%)	76 (20.43%)	2.75 (1.87–4.05)	<0.0001	1.96 (1.23–3.12)	**0.004**
SRF+IRF	66 (35.48%)	54 (14.52%)	3.24 (2.14–4.91)	<0.0001	2.10 (1.29–3.42)	**0.003**
PED	118 (63.44%)	204 (54.84%)	1.43 (1.00–2.05)	0.053	0.94 (0.53–1.65)	0.81
PED with SRF or IRF	184 (98.92%)	365 (98.12%)	1.76 (0.36–8.57)	0.48	0.84 (0.17–4.22)	0.82

OR, odds ratio.

a Analysis controlled by age and baseline CST.

The exclusive effects of each anti-VEGF agent on both fluid fluctuation metrics and clinical outcomes were analyzed. We found that the mean FI from month 3 to 12 in patients receiving aflibercept monotherapy during the same period was significantly less than those treated with bevacizumab monotherapy (48.8 vs. 63.4). Nevertheless, this did not translate to clinically significant differences in mean letter improvement at 12 months (7.1 vs 5.8 letters; *P* = 0.96) (Supplementary Table S2). Patients receiving ranibizumab monotherapy after the initial loading phase were excluded from the analysis because of the inadequate sample size for calculation. Similarly, the analysis using the same model at 24 months was limited by significantly fewer patients continuing the same anti-VEGF agents.

## Discussion

The present study demonstrated that eyes presenting with SRF concurrent with IRF likely exhibited unstable CST after 3 loading doses of bevacizumab treatment. Our results partly correspond with a previous observations reporting that every 100-µm increment in baseline CST was associated with an increase in CST SD by 24, which further translated to worsening 24-month visual outcomes.^10^ Theoretically, accumulating fluid in subretinal space and neuroretinal compartments represents disease chronicity or more extensive involvement of MNV where physiological hemostasis of blood retinal barriers has been disrupted.^16^ In case of insufficient treatment, such longstanding vascular leakage could outweigh rates of fluid absorption by the retinal pigment epithelium and accumulatively produces stagnant exudates above the external limiting membrane barrier and subretinal fibrosis; These may decelerate responses to anti-VEGF medications and subsequent fluctuating foveal thickness. The fact that PED did not link to the CST variation (Table 3) may reflect the suboptimal responses of PED to anti-VEGF treatment, particularly when bevacizumab was the most selected medication.^17^^–^^20^ Of note, although most independent analyses were corrected for possible confounders like polypoidal lesions or type III MNV, some factors relating to the acute response, such as the type of lesion, lesion size, and other fundus fluoresceine characteristics, were not addressed.

To extrapolate the results to general practice, it has become apparent that unstable macular thickness in patients continuing anti-VEGF therapy reflects incomplete or suboptimal treatment, which consists of either ineffectiveness of anti-VEGF agents or insufficient drug durability. Owing to individual variations among patients, some patients may receive relatively inadequate treatment frequency compared with their high disease activity, and therefore require more explicit attention by their physicians. As such, when combined with numerous factors used to make treatment decisions in clinical practice, eyes possessing high-risk features for unstable CST should trigger physicians not only to adhere strictly to the treatment protocol,^15^ but also to consider switching the treatment regimen or adopting an alternative approach with better drug durability.^21^^–^^23^ Referring to the study results, a decrease in the mean FI was observed in patients receiving aflibercept monotherapy during the maintenance phase, compared with those without regimen switching. Nonetheless, this difference did not yet transfer to a significant visual benefit over the short-term period of 12 months (Supplementary Table S2).

To validate the potential use of the FI, the fact that the metric includes any magnitude in CST changes occurring between each visit is another difference between our parameter and the previously reported Fluctuation Score,^14^ which excludes CST changes of <50 µm from the score calculation. We speculate that such small changes in CST during each visit may be clinically insignificant; however, these repeating instances can have a significant impact on eventual outcomes.

The correlations between visual outcomes and the FI calculated from pooled data showed that the greater the patients’ fluid fluctuation degree (from the low to the high fluctuation group), the less visual improvement they were likely to achieve at 24 months (Table 3). Although not statistically significant, such a negative correlation with a degree–response gradient may support the validity of the FI as a suitable outcome parameter for applying in the future real-world study involving retinal fluid variability. Specifically, the FI presented a stronger negative correlation with BCVA improvement than CST SD. Thus, after a 24-month course of treatment, the FI may be considered as an indirect method to assess MNV activity, treatment adequacy, and responses to anti-VEGF medications in each patient. The fact that a 12-month FI value of <27.8 was associated with a mean 2-year BCVA of ≥20/63 may aid physicians to plan further on individualized treatment for patients, particularly in those with unexpectedly substandard visual outcomes.

To compare the baseline features of our real-world setting with those from clinical trials, the median SD of 1-mm CST in our cohort (40.6) was close to that of foveal center point thicknesses (40.2) drawn from the IVAN study.^5^ Regarding follow-up adherence, we speculate that the inability of patients to maintain frequent visits was partly due to the relatively short half-life duration of bevacizumab, the predominant drug used in Thailand.^24^^–^^26^ In addition, the majority of our study eyes had PCV, which has shown suboptimal responses to bevacizumab with a polypoidal regression rate of only 24%.^27^ Hence, those eyes potentially ended up having some stages of fibrosis, which may have affected physicians’ decisions to prescribe treatment, despite the presence of retinal fluid, owing to its poor visual benefits.^28^ Taken together, this could result in a substantial decrease in the number of injections after 12 months.

In addition to foveal thickness, this study emphasizes the importance of its variability during postloading anti-VEGF treatment as an independent factor associated with poor visual outcomes. Despite the indifferences in the final CST between eyes in the low and moderate fluctuation groups at 24 months, patients with moderate CST fluctuation achieved less BCVA (Table 1, Fig. 1). Deteriorated visual results in these cases may be a product of irregularly emerging retinal fluid resulting in chronic intermittent, albeit, progressive structural changes of the outer retina, which include, but are not limited to, outer retinal tubulation and subretinal fibrotic scarring (Fig. 3). Our results correspond with those from post-hoc analyses of the Comparison of AMD Treatment Trial (CATT) and the Inhibition of VEGF in Age-Related Choroidal Neovascularization (IVAN) randomized clinical trial showing that large variations in foveal thickness complicating nAMD treatment were associated with worse BCVA and the development of fibrosis.^5^ Such changes in photoreceptors act as a barrier against fluid from MNV migrating across the retinal pigment epithelium to the subretinal space. Ultimately, the amount of SRF or IRF decreases in parallel with decreasing CST and visual deterioration.^29^ Of note, our study's analysis did not account for the factor of subretinal hemorrhage, which presented in a small proportion of study eyes (11.7%).^30^

Regarding the degrees of visual improvement, most previous studies have reported approximately 6 to 10 Early Treatment Diabetic Retinopathy Study letter differences in 24-month BCVA between patients possessing first quartile and those with fourth quartile of macular thickness SD.^5^^,^^11^^–^^13^ Notably, the greater differences in such values reported in our study (14 letters) may result not only from dissimilar methodology, where the patients were split into three parts instead of quartiles, but variation in the adjusted parameters, such as the presenting macular thickness.^10^^,^^12^ What distinguishes our study from the others is that we considered baseline CST as one of the adjusted covariates before calculating visual results.^5^^,^^12^^,^^13^

Hypothetically, MNV accompanying thin SRF may be an imperfect yet critical compensatory mechanism for degenerative macula associated with outer retinal hypoxia. Several studies found that SRF was stable after appropriate proactive initiation of anti-VEGF appears benign on visual outcomes and could even be protective against the incident geographic atrophy.^18^^,^^31^^–^^34^ Hence, one may tolerate a slight degree of persistent SRF if visual acuity is not compromised, whereas persistent IRF should be treated intensively until the patient is clinically stable. Referring to our cohort, treatment decision was highly influenced by any amount of SRF or IRF despite the concept of SRF tolerance, and less so in non-PCV eyes presenting with stationary PED without SRF or IRF. This finding may reflect real-world practice, where low rates of follow-up adherence were expected,^35^ whereas PED acceptance may be due to a dissociation between variation in magnitude of PED or its resolution and visual benefits.^17^^–^^20^^,^^36^^,^^40^

Our study has limitations associated with the retrospective nature and collection of data from routine practice. These limitations include variability in treatment switching criteria and heterogenous medications used. However, no significant differences were observed in either the injection number of each anti-VEGF brand (Table 2) or the total numbers of injections among the three groups (Table 1). Moreover, the factors mentioned above that could influence disease progression and responses to anti-VEGF therapy were not included in the analysis.^37^^,^^38^ Specifically, despite the lack of differentiation between type 1 and type 2 MNV, the study's results were partially governed by qualitative factors, such as the proportions of eyes with polypoidal lesions that did not diverge across the three study groups. It is to emphasize that the study population analyzed is exclusively Asian and that the results obtained may not be generalized to the entire population worldwide. More important, individuals aged 40 to 50 years included in this study may not generally be representative of typical AMD, particularly in case of pachychoroid neovasculopathy or polypoidal lesions since their pathogenesis differs from MNV arising from drusen-associated AMD.^39^^,^^40^ Finally, anti-VEGF nonresponders with persistent macular edema who rarely had anatomic improvement may represent as cases in the low fluctuation group. To avoid misinterpretation related to such paradoxical consequences, the analysis was controlled by the total number of injections and baseline retinal thickness. A future study may explore the integration of the FI into a software of OCT devices, or its application in artificial intelligence to help the clinician in assessing the best interval of treatment or the decision of switching therapy. We also recommend to investigate further on predictors of macular thickening variability at month 3 after a loading phase.

## Conclusions

Unstable macular thickening represented by a large FI is associated with inferior visual outcomes during the maintenance phase of nAMD treatment. Compared with the SD of CST values, this study supports the value of the FI as one of the parameters for representing therapeutic adequacy or individuals’ responses after a course of anti-VEGF therapy. Among the retinal fluid subtypes, the presence of IRF with concurrent SRF at the baseline visits showed the strongest association with foveal thickness instability during the maintenance phase of treatment.

## Supplementary Material

Supplement 1
