# Supplementary material for: Clinical Correlation of Retinal Fluid Fluctuation Represented by Fluctuation Index in Wet Age-Related Macular Degeneration: TOWER Study Report 2
Source: Transl Vis Sci Technol. 2023 Oct 3;12(10):2. doi: 10.1167/tvst.12.10.2 (PMC10552872; doi:10.1167/tvst.12.10.2)
Supplement: Supplement 1 [file tvst-12-10-2_s001.pdf]

Supplement

List of Tables **Page**

Table S1 Multiple Logistic Regression Analyzing Baseline Characteristics for High CST Fluctuation over 24 months in Eyes with Neovascular AMD…………………………………1

Table S2 12-month Clinical Outcomes and Retinal Fluid Fluctuation Metrics Categorized by Anti-VEGF Medications in Patients Receiving the Same Treatment Regimen…………2

Table of figures **Page**

Figure S1 A simulated model to explore the association between fluid fluctuation metrics (standard deviation of central subfield thickness and Fluctuation Index) and different patterns of retinal fluid fluctuation over the course of anti-VEGF treatment........................3

Figure S2 Mean changes in 1-mm central subfield thickness (with 95% confidence interval) characterized by Fluctuation Index...4

Table S1 Multiple Logistic Regression Analyzing Baseline Characteristics for High CST Fluctuation over 24 months in Eyes with Neovascular AMD

	High Fluctuation (186 eyes)	Low to Moderate Fluctuation (372 eyes)	Odds Ratio (OR)			
			Crude OR (95% CI)	*P*	Adjusted OR* (95% CI)	*P**
Age			1.02 (1.00 - 1.04)	0.06	1.01 (0.99 – 1.03)	0.33
Baseline LogMAR BCVA (every 1.0 increment)			1.70 (1.25 – 2.31)	0.0007	1.17 (0.80 – 1.72)	0.41
Baseline CST ≥405 µm (%)	85 (45.7%)	81 (21.7%)	3.51 (2.28 – 5.42)	<0.0001	3.27 (2.08 – 5.13)	<0.0001
Use of Bevacizumab (%)	152 (81.7%)	287 (77.1%)	1.32 (0.85 – 2.06)	0.21	1.12 (0.65 – 1.96)	0.68
Diagnosis of PCV (%)	111 (59.6%)	214 (57.5%)	1.09 (0.76 – 1.56)	0.62	1.24 (0.76 – 2.03)	0.38

CST = central subfield thickness; AMD = age-related macular degeneration; BCVA = best-corrected visual acuity; PCV = polypoidal choroidal vasculopathy.

*Adjusted for all variables.

Table S2 Clinical Outcomes and Retinal Fluid Fluctuation Metrics Categorized by Anti-VEGF drugs in Patients Receiving the Same Treatment Regimen from Month 3 to 12.

	Aflibercept (58 eyes)	Bevacizumab (263 eyes)	*P*-value*
Mean CST-SD from month 3 to 12 (95% CI)	39.7 ± 46.1 (27.6 - 51.8)	48.5 ± 49.0 (42.6 - 54.45)	0.12
Mean Fluctuation Index from month 3 to 12 (95% CI)	48.8 ± 49.6 (35.8 - 61.9)	63.4 ± 58.9 (56.3 - 70.6)	0.04
**Mean BCVA (ETDRS letters)**			
Baseline	48.6 ± 25.6	42.4 ± 27.4	0.16
12-month	55.7 ± 23.7	48.2 ± 28.1	0.098
12-month letter gain (95% CI)	7.1 ± 18.5 (2.2 - 11.9)	5.8 ± 22.2 (3.1 - 8.5)	0.96
**Mean CST (μm)**			
Baseline	441 ± 212	428 ± 193	0.72
12-month	273 ± 86	300 ± 127	0.36

AMD = age-related macular degeneration; VEGF = vascular endothelial growth factor; CST = central subfield thickness; SD = standard deviation; CI = confidence interval; BCVA = best-corrected visual acuity; ETDRS = early treatment diabetic retinopathy study.
*P-value based on Wilcoxon rank sum test

Figure S1 A simulated model to explore the association between fluid fluctuation metrics (standard deviation of central subfield thickness and Fluctuation Index) and different patterns of retinal fluid fluctuation over the course of anti-VEGF treatment.

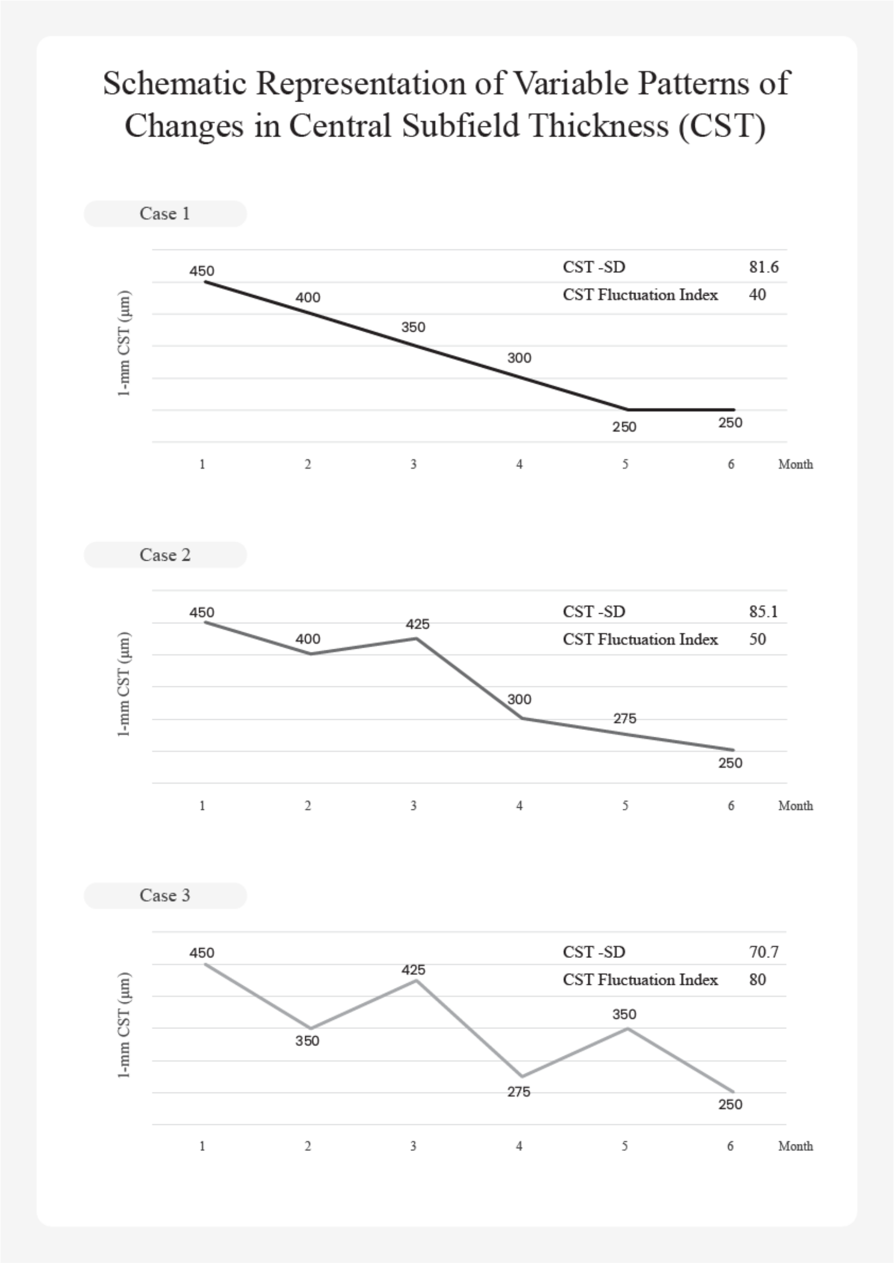

The model showed that a patient with an obvious zigzag pattern of retinal thickness has the lowest SD value (bottom panel).

Figure S2 **Mean changes in 1-mm central subfield thickness (with 95% confidence interval) characterized by Fluctuation Index.**

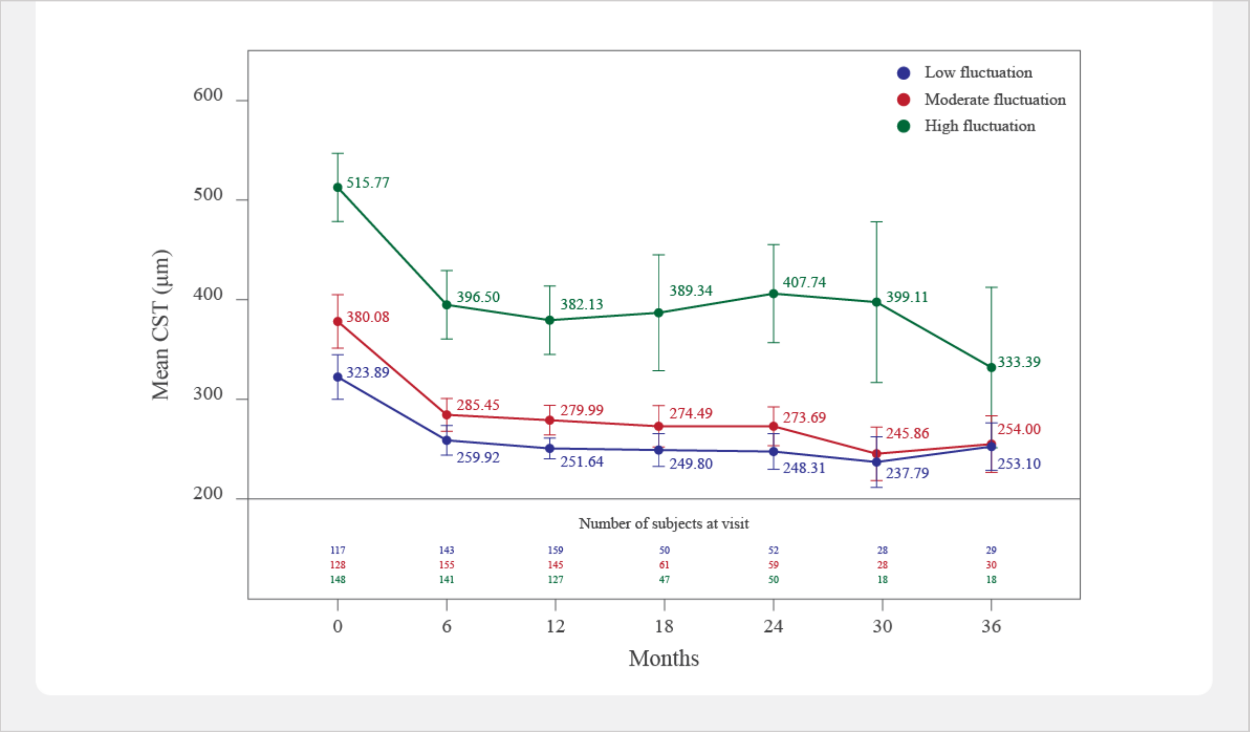

There was a trend towards CST reduction throughout the treatment course in all groups. At 24 months, no differences in CST were detected between the low and moderate fluctuation group (P = 0.71). Although we did not systematically examine the effects of retinal fluid fluctuations on most clinical outcomes after month 24, the mean CST became statistically indifferent among the three groups at month 36 (Global P = 0.67).
